# The Role of Lateral Internal Sphincterotomy in Haemorrhoidectomy: A Study in a Tertiary Care Center

**DOI:** 10.7759/cureus.15630

**Published:** 2021-06-13

**Authors:** Shashikanth Vijayaraghavalu, Guru Prasad R, Sathish Rajkumar

**Affiliations:** 1 General Surgery, ACS Medical College Hospital, Chennai, IND; 2 General Surgery/Surgical Oncology, ACS Medical College Hospital, Chennai, IND

**Keywords:** lateral internal sphincterotomy, hemorrhoids, haemorrhoidectomy, incontinence, anal stenosis, anal fissure

## Abstract

Background

Hemorrhoids are a common condition that presents with bleeding per rectum, pain at rest and defecation, mucosal discharge, and prolapse. Surgical hemorrhoidectomy is the treatment method of choice for Grade 3 and Grade 4 hemorrhoids. Hemorrhoidectomy is associated with postoperative pain and no single surgical technique has been proved to significantly reduce the pain. We analyzed in our study the effect of lateral internal sphincterotomy with hemorrhoidectomy on postoperative pain, anorectal function, and retention of urine after the Milligan and Morgan technique.

Methods

This randomized, prospective, and comparative study included 200 Grade 3 and Grade 4 hemorrhoids patients who were scheduled for surgical management. The patients were classified randomly into two groups with an equal number of participants: Group A underwent Milligan & Morgan open hemorrhoidectomy and Group B underwent lateral internal sphincterotomy (LIS) in addition to Milligan and Morgan open hemorrhoidectomy. Postoperative pain was recorded using the Visual Analog Scale (VAS) score for up to 48 hours. Postoperative bleeding, urinary retention, and bowel and gas incontinence were noted. Long-term follow-up at six and 24 months for anal stenosis, anal fissure, incontinence, and recurrence was also noted.

Results

Patients who underwent LIS showed a significant reduction in postoperative pain at 12 hours (p=0.0008*), 24 hours (p=0.000*), and 48 hours (p=0.003*); the time taken to request rescue analgesia was similar between the two groups (p=0.07). Side effects, such as postoperative bleeding and urinary retention, were significantly lower after LIS (p=0.001* and p=0.01*, respectively), and gas incontinence was significantly higher after LIS (p=0.002*). The long-term outcomes of anal fissure were significantly higher without LIS at six months (p=0.02*) and 24 months (p=0.04*) and those of anal stenosis were significantly higher without LIS at six months (p=0.04*).

Conclusions

From our study, we conclude that postoperative pain, bleeding, and urinary retention were significantly lower after LIS, and gas incontinence was transient. The long-term outcomes, which included anal stenosis and anal fissure, were significantly lower after LIS. However, bowel and gas incontinence and recurrence were not altered. Therefore, we conclude that the addition of LIS to hemorrhoidectomy improves patient outcomes in terms of postoperative pain and anorectal function.

## Introduction

Hemorrhoids are formed by the downward displacement of dilated submucosal vascular anal cushions, commonly located at 3, 7, and 11 o'clock in the anal canal. It is a common condition which presents with complaints of bleeding per rectum, pain at rest and defecation, mucosal discharge, and prolapse. Surgical hemorrhoidectomy is the treatment method of choice for Grade 3 and Grade 4 hemorrhoids [1,2].

The surgical techniques employed include the Milligan and Morgan technique, which is most commonly performed, the Ferguson technique, stapled hemorrhoidectomy, the technique using a harmonic scalpel or ligasure, and Doppler-guided hemorrhoidal artery ligation. The early complications after surgery include postoperative pain, primary hemorrhage, and retention of urine [3].

Hemorrhoidectomy is associated with postoperative pain, and no single surgical technique has been proved to significantly reduce the pain [4]. The pain following hemorrhoidectomy may be due to anal packing, urinary retention, wound edema, or increased tone due to spasm of the internal sphincter [5]. Various invasive and non-invasive techniques have been employed to relieve the spasm such as topical nitroglycerin (NTG), calcium channel blocker (CCB), Lord’s dilatation, and lateral sphincterotomy. Each of these measures has its side effects.

We analyzed in our study the effect of lateral internal sphincterotomy with Milligan and Morgan hemorrhoidectomy on postoperative pain, anorectal function, and retention of urine.

## Materials and methods

This randomized prospective comparative study was performed in ACS Medical College Hospital after obtaining institutional ethical committee clearance. The following patients were enrolled in the study: patients with clinical and investigatory support for the diagnosis and willingness for the surgical management of Grade 3 and 4 hemorrhoids. Any patient with the following was excluded from the study: inflammatory bowel disease, fissure, recurrent hemorrhoids, fistula, malignancy, cirrhosis, and portal hypertension, pregnancy, and age < 20 and > 60 years. All patients enrolled in the study were explained about the study procedure and their informed written consent was taken. The study comprised 200 patients of Grade 3 and 4 hemorrhoids who were randomly divided into two groups of 100 each, named Group A and Group B. In Group A, only Milligan and Morgan open hemorrhoidectomy was done, and in Group B, lateral internal sphincterotomy (LIS) was done in addition to Milligan and Morgan open hemorrhoidectomy.

Preoperatively, patients in both groups were optimized. Patients in both groups were started on metronidazole therapy for five days, along with oral liquid paraffin. All patients in the study were prescribed local application of glycerine and magnesium sulfate soaked dressings to reduce edema and relieve pain. Patients were also advised to take a Sitz bath thrice a day and were taught to maintain proper anal hygiene. All patients were given an enema early morning on the day of surgery. Patients in both groups received spinal anesthesia.

After spinal anesthesia, and after the optimal level of block was achieved, the patients were put in lithotomy position, parts painted, and draped. A per rectal examination and preliminary proctoscopy were done. The anal skin was held with Allis forceps and retracted first at the 7 o'clock position. The hemorrhoidal mass was visualized and held with artery forceps. A V-shaped incision was made at the skin from the anoderm up to the anal mucosa, followed by gentle dissection. The hemorrhoid mass was separated from the internal anal sphincter till the apex of the pedicle was reached. The hemorrhoid pedicle was ligated using vicryl (absorbable suture) and excised in toto. The bare area was left open. A similar procedure was repeated first at 3 o'clock position, and finally at the 11 o'clock position. Complete hemostasis was obtained. An anal pack was kept which was soaked in liquid paraffin and povidone-iodine.

For those in Group B, in addition to the Milligan and Morgan technique, a lateral internal sphincterotomy was also done. Intersphincteric groove was palpated. A 1 cm lateral incision made at 3 o'clock position near the anal verge. Gentle dissection was done along the submucosa till the internal sphincter was reached. The lower free end of the internal sphincter was hooked out using artery forceps and divided. Complete hemostasis was obtained and the wound was left open. The intraoperative bleeding during the surgery was kept to a minimum using cautery and gentle dissection.

Postoperatively, all the patients were kept nil per oral for six hours after surgery and later started on a liquid diet, followed by a soft, high-fiber diet. All the patients in Group A and Group B were routinely given injection paracetamol 1 g intravenously (IV) on the night of surgery. If the patients complained of pain prior to this, then injection tramadol 100 mg IV was given as rescue analgesia. The anal pack was removed in both groups after six hours postoperatively. The patients were also advised on the need for early ambulation and a sitz bath postoperatively to minimize the chances of infection.

The primary objective of the study was pain relief after surgery in the postoperative period. The pain was assessed by using the Visual Analog Scale (VAS) at 12, 24, and 48 hours with a rating ranging from 0-10: 0 - no pain, 1-3 - mild pain, 4-6 - moderate pain, 7-10 - severe pain. The time taken for the first request of rescue analgesia was also noted. The secondary objective included postoperative complications. Factors taken into account included postoperative incidence of bleeding, urinary retention, and bowel and gas incontinence until 48 hours.

The observation and data collection of bleeding was assessed subjectively into groups based on the amount of bleeding. They were grouped as: None - no bleeding; Confined - minimal loss/occasional episodes during defecation (<20 ml of blood); Moderate - frequent episodes during defecation (>20 ml of blood); Severe - persistent bleeding even without defecation with fall in hemoglobin levels (<10 g/dl requiring hematinics or <7 g/dl requiring blood transfusion).

Both the groups were observed for the inability to void urine under voluntary control. For those who failed to void voluntarily, bladder catheterization was done. Percentage of patients who required bladder catheterization for urine drainage was noted. Incontinence was assessed by Wexner's incontinence score for solid, liquid, and gas. Short-term and long-term follow-ups were done at six and 24 months after surgery to rule out late complications like anal fissure, anal stenosis, bowel or gas incontinence, and recurrence.

Statistics

Data were analyzed using the Statistical Package for Social Sciences (SPSS); continuous variables were analyzed using unpaired t-test for two means, and categorical data were analyzed using Chi-square test. Data were expressed as mean±SD and as percentages where appropriate. P-value <0.05 was considered statistically significant.

## Results

In the present study, both groups were comparable in terms of age, weight, sex, and grade of disease (Table 1).

**Table 1:** Demographic data of patients in the two study groups

	Age (mean±SD)	Weight (mean±SD)	Sex (no. of cases)	Grade of disease 3/4 (no. of cases)
Group a	42.15±12.4	77.73±6.9	M(60) f(40)	46/54
Group b	41.03±10.9	76.83±6.2	M(60) f(40)	42/58
P-value (two-sample t-test)	0.49 (ns)	0.33 (ns)	-	0.52 (ns)

The VAS score was assessed at 12 hours, 24 hours, and 48 hours postoperatively. The mean VAS score in Group A was 6.12 at 12 hours, 5.61 at 24 hours, and 4.04 at 48 hours. The Mean VAS score in Group B was 5.1 at 12 hours, 3.4 at 24 hours, and 3.2 at 48 hours. The VAS score was statistically significant at 12, 24, and 48 hours. The p-values were 0.0008* (12 hours), 0.000* (24 hours), 0.003* (48 hours) (Table 2). The mean time taken for the first requirement for rescue analgesia in Group A was 191.6 mins, and in Group B, it was 189.7 mins, which were not statistically significant. The p-value was 0.07 (ns) (Table 2).

**Table 2:** Postoperative VAS scores and rescue analgesia requirement * significant; ns: not significant; VAS: Visual Analog Scale

VAS score post-operatively	Group A (mean±SD)	Group B (mean±SD)	P-value (two-sample t-test)
12 hours	6.12±2.3	5.1±1.9	0.0008*
24 hours	5.61±1.2	3.4±1.1	0.000*
48 hours	4.04±1.1	3.2±2.5	0.003*
Time taken for the first request of rescue analgesia	191.6±1.58	189.7±10.5	0.07(ns)

Postoperative bleeding in Group A was confined in 76 patients and moderate bleeding occurred in 14 patients. In Group B, confined bleeding occurred in 72 patients and moderate bleeding in three patients. None of the study patients had severe bleeding. Ten patients in Group A had no bleeding, and 25 patients in Group B had no bleeding. The incidence of postoperative bleeding was statistically significant between the groups (p-value = 0.001*) (Table 3). Urinary retention requiring catheterization was seen in 17 patients in Group A and six patients in Group B, which was statistically significant (p=0.01*) (Table 3). Gas incontinence occurred in 9 patients in group B and none in group A which was statistically significant (p=0.002*) (Table 3). None of our patients had bowel incontinence in the immediate postoperative period.

**Table 3:** Postoperative complications in both groups * significant

Postoperative complications	Group A (n=100) (no. of cases)	Group B (n=100) (no. of cases)	P-value (Chi-square test)
Bleeding: None	10	25	0.001*
Confined	76	72	
Moderate	14	3	
Severe	0	0	
Urinary Retention requiring catheterization	17	6	0.01*
Bowel incontinence	0	0	-
Gas incontinence	0	9	0.002*

Follow-up of our patients was done at six months. Of the 200 study patients, 186 patients were followed up: 92 in Group A and 94 in Group B. Five patients had an anal fissure and four had anal stenosis in Group A. In Group B, none had an anal fissure or anal stenosis. This was statistically significant with p=0.02* and 0.04*, respectively (Table 4). No patient had a recurrence at six months in both groups. Two patients in Group B had gas incontinence and one had bowel incontinence at six months, which was not statistically significant - p-values 0.15 (ns) and 0.32 (ns), respectively (Table 4).

**Table 4:** Follow-up of patients at six months and 24 months * significant; ns: not significant

	After 6 months			After 24 months		
	Group A (n=92) patients (%)	Group B (n=94) patients (%)	P-value (Chi-square test)	Group A (n=81) patients (%)	Group B (n=77) patients (%)	P-value (Chi-square test)
Anal fissure	5(5.4%)	0	0.02*	4(4.9%)	0	0.04*
Anal Stenosis	4(4.3%)	0	0.04*	3(3.7%)	0	0.08(ns)
Bowel incontinence	0	1(1.06%)	0.32(ns)	0	0	-
Gas incontinence	0	2(2.12%)	0.15(ns)	0	0	-
Recurrence	0	0	-	6(7.4%)	2(2.5%)	0.16(ns)

A long-term follow-up at 24 months was done. Of the 200 patients, 158 patients were followed up: 81 in Group A and 77 in Group B. Four patients had an anal fissure in Group A and none in Group B, which was statistically significant p=0.04* (Table 4). Three patients had anal stenosis in Group A, in Group B, none had anal stenosis, p=0.08(ns) (Table 4). Six patients in Group A and two in Group B had recurrence, which was not statistically significant, p=0.16 (ns) (Table 4). None of the patients had bowel or gas incontinence at 24 months.

## Discussion

Postoperative pain after haemorrhoidectomy is attributed to high anal canal pressure due to increased tone of the internal sphincter. Eisenhammer was the first to discover that spasm of internal sphincter is responsible for post-haemorrhoidectomy pain [6]. The hypothesis of haemorrhoidectomy with lateral internal sphincterotomy reducing postoperative pain is still controversial. Several studies have compared the postoperative outcomes of haemorrhoidectomy alone and haemorrhoidectomy with lateral internal sphincterotomy.

Kanellos et al. in their study of 78 patients with Grade 4 hemorrhoids showed a statistical difference in pain scores after first defecation and one week after surgery [4]. The immediate pain scores at 12 hours were not significantly different between the groups. Diana et al. in their study with 699 patients reported postoperative pain in 192 patients, and it was the most frequent postoperative complication - 23.03% - and the number of patients who had postoperative pain decreased significantly when lateral internal sphincterotomy was done, going down from 28.8% to 10.45%. They concluded that there was significant pain reduction only in the first postoperative period and not on medium- long-term follow-up [7].

In our study, the mean pain scores at 12, 24, and 48 hours were significantly lower in the group that underwent LIS in addition to haemorrhoidectomy. This is contrary to a study by Khubchandani who reported no statistical difference in postoperative pain, but this was attributed to performing LIS distal to the dentate line, terminating at the inter-sphincteric groove [8].

The time taken for the first request of analgesia was similar in both groups and did not show a statistical difference - similar to the study by Khubchandani [8]. Galizia et al. in their randomized study found a significant difference in pain scores and analgesic requirement between the two groups. However, they suggested that it was justifiable only when preoperative anorectal manometry showed high anal pressure [9].

The incidence of postoperative bleeding in our study was significantly lower in patients with LIS, whereas, among patients without LIS, 14 complained of moderate bleeding. The addition of LIS had no increased incidence of postoperative bleeding. This could be attributed to reduced straining during defecation due to relaxed anal tone and reduced spasm. Postoperative bleeding may cause anemia very rapidly in some patients. None of our patients experienced any adverse effects related to bleeding. Postoperative bleeding may be due to lack of thrombus or its expulsion or dissociation, and reabsorption of the transfixed stitch. Depending on the study considered, postoperative bleeding frequency varied between 0.6% and 10% [10,11].

Urinary retention incidence in the postoperative period requiring catheterization in our study was 17% in patients with haemorrhoidectomy and 6% in patients with LIS and haemorrhoidectomy. Toyonaga et al. [12] in their study reported an incidence of 21.9%.

Postoperatively, patients having LIS may have a significantly increased risk of incontinence as suggested by some authors. Studies have also shown that sphincterotomy is accompanied by a low incidence of transient incontinence [13,14]. In our study, nine patients who underwent LIS had gas incontinence in the immediate postoperative period. None of our patients had bowel incontinence in the immediate postoperative period irrespective of the surgery performed.

We followed up our patients at six months and 24 months for anal fissure, anal stenosis, and bowel and gas incontinence. Patients developing anal stenosis usually have complaints of either anal pain, constipation, or bleeding [15,16]. Anal stenosis is believed to be reduced in LIS, whereas it is a rare serious complication of haemorrhoidectomy. The majority of studies reported no anal stenosis after LIS; since LIS shows a preventive role against stenosis, its routine inclusion with haemorrhoidectomy may be justified [17,18]. In our study, five patients and four patients who did not undergo LIS developed anal fissure at six and 24 months, respectively. Four and three patients had anal stenosis at six and 24 months, respectively - this may be due to increased anal tone caused by healing and fibrosis. Two patients who underwent LIS complained of gas incontinence and one patient complained of bowel incontinence.

Haemorrhoidal recurrence occurs in around 2-8% of patients post-surgery [19]. Earlier, the incidence was around 14%, as mentioned by Goligher in their study [20]. In our study, at 24-month follow-up, eight patients had complaints of recurrence, predominantly at the primary site. The incidence was higher in patients who underwent only open haemorrhoidectomy without LIS (7.4%) and was significantly lower in patients with LIS (2.5%).

## Conclusions

From our study, we conclude that postoperative pain, bleeding, and urinary retention were significantly lower after LIS, and gas incontinence was transient. The long-term outcomes, which included anal stenosis and anal fissure, were significantly lower after LIS. However, bowel and gas incontinence and recurrence were not altered. Therefore, we conclude that the addition of LIS to hemorrhoidectomy improves patient outcomes in terms of postoperative pain and anorectal function.
